# The role of a modified emergency nursing pathway based on the shared decision-making model in alteplase thrombolysis for acute ischemic stroke: A study on establishing an emergency department nursing system

**DOI:** 10.1097/MD.0000000000049255

**Published:** 2026-06-26

**Authors:** Haixia Liu

**Affiliations:** a Emergency Room, Bozhou People’s Hospital, Bozhou, Anhui Province, China.

**Keywords:** acute ischemic stroke, alteplase, emergency department, modified emergency nursing pathway, shared decision-making model

## Abstract

This study aimed to analyze the effect of a modified emergency nursing pathway based on the shared decision-making model during alteplase thrombolysis for acute ischemic stroke and provide a reference for establishing a nursing system in the emergency department. Eighty patients with acute ischemic stroke treated with alteplase thrombolysis in our hospital from October 2023 to August 2024 were selected as the study subjects. They were divided into a control group (receiving conventional emergency care) and an observation group (receiving the modified emergency nursing pathway based on the shared decision-making model), with 40 cases in each group. The total effectiveness rate, triage time, thrombolysis waiting time, rescue time, complication rate, National Institutes of Health Stroke Scale scores, and Activities of Daily Living scale scores before and after thrombolysis were compared between the 2 groups. The modified Rankin Scale scores at discharge were obtained through follow-up. Compared with the control group, the observation group had a significantly higher total effectiveness rate and a lower complication rate (both *P* < .05). Triage time, thrombolysis waiting time, and rescue time were significantly shorter in the observation group (all *P* < .05). After thrombolysis, the observation group showed lower National Institutes of Health Stroke Scale scores, higher Activities of Daily Living scores, and lower modified Rankin Scale scores at discharge (all *P* < .05). After adjustment for potential confounders, these associations remained statistically significant, indicating that the modified emergency nursing pathway was independently associated with improved clinical and process outcomes. The application of the modified emergency nursing pathway based on the shared decision-making model during alteplase thrombolysis for acute ischemic stroke can improve treatment efficiency and efficacy, reduce complications, enhance neurological function and prognosis, and increase the ability in daily activities. It is worthy of adoption. However, this study was conducted in a single center with a relatively small sample size, which may limit the generalizability of the findings. Future multicenter studies with larger samples are needed to further validate these results.

## 1. Introduction

Acute ischemic stroke is a common critical emergency condition characterized by sudden onset, rapid progression, and high risks of disability and mortality.^[1]^ According to relevant data,^[2]^ the prevalence of acute ischemic stroke in China has increased from 117/1,00,000 in 2005 to 145/1,00,000 as of 2019. Failure to receive timely treatment can lead to language impairments and hemiplegia, further increasing the burden on families and society as a whole. Early alteplase thrombolysis can effectively recanalize occluded blood vessels, alleviating tissue ischemia and hypoxia, and is currently an effective treatment for this condition.^[3]^ However, thrombolytic therapy has strict time requirements. If treatment is not initiated within the 4.5-hour golden window, the success rate significantly decreases, while the risks of disability and mortality correspondingly increase.^[4]^ Therefore, standardizing and improving emergency care alongside treatment plays a crucial role in enhancing emergency efficiency. The management of acute ischemic stroke requires multiparty collaboration and communication. However, several issues and challenges persist, such as non-standardized processes, uneven resource distribution, and irrational decision-making, which can directly impact rescue efficiency and patient outcomes.^[5]^

The shared decision-making model is a collaborative decision-making approach involving multiple stakeholders. A study by Skolarus et al^[6]^ found that emergency care under this model, which incorporates information exchange, risk assessment, value considerations, and treatment option selection, helps formulate optimal emergency plans. This ensures more individualized and patient-centered emergency care, thereby improving the efficiency and effectiveness of acute ischemic stroke management. However, clinical reports on the application of a modified emergency nursing pathway based on the shared decision-making model in thrombolysis for acute ischemic stroke remain scarce. Thus, this study analyzes the role of a modified emergency nursing pathway under the shared decision-making model during alteplase thrombolysis for acute ischemic stroke, aiming to provide guidance for developing clinical emergency nursing protocols.

## 2. Materials and methods

### 2.1. Study subjects

This study was approved by the Ethics Committee of Bozhou People’s Hospital. Eighty patients with acute ischemic stroke treated with alteplase thrombolysis at our hospital from October 2023 to August 2024 were selected as the study participants. This was a retrospective observational study based on routinely collected medical records. Eligible patients were identified from the hospital electronic medical record system. Data were obtained from emergency triage records, nursing records, thrombolysis pathway records, laboratory and imaging reports, and discharge summaries. A standardized data abstraction form was used. Data were independently extracted by 2 trained investigators. Discrepancies were resolved by discussion, and if necessary, adjudicated by a senior clinician. Patients were classified into the control group or observation group according to the emergency nursing pathway documented in the medical records during the index hospitalization.

Inclusion criteria were as follows: diagnosis of acute ischemic stroke confirmed by signs, symptoms, and cranial computed tomography (CT) examination, meeting the diagnostic criteria for the disease^[7]^; first episode of stroke; time from onset to hospital admission within 4.5 hours; meeting the indications for thrombolytic therapy.

Exclusion criteria included: death before thrombolysis could be initiated after admission; hemorrhagic cerebral infarction; history of cerebral hemorrhage or active bleeding; presence of interfering conditions such as severe hepatic, renal, or cardiac diseases, psychiatric disorders, or malignancies.

This study was approved by the hospital’s Ethics Committee, and informed consent was obtained from all patients.

The sample size was determined based on preliminary clinical observations and similar studies reported in the literature. Considering feasibility and available patient resources, 80 patients (40 per group) were included in this study. Although the sample size was relatively limited, it was considered sufficient to detect clinically meaningful differences between the 2 groups.

### 2.2. Methods

#### 2.2.1. Treatment method

All patients received symptomatic treatment based on their condition, including measures to lower blood pressure, reduce intracranial pressure, and correct water-electrolyte imbalances. Alteplase thrombolysis was administered as follows: alteplase was dosed at 0.9 mg/kg, with a maximum dose of 90 mg. After determining the dose, it was dissolved in an equivalent volume of solvent. First, 10% of the total dose was administered intravenously as a bolus, and the remaining portion was infused via an infusion pump over 60 minutes.

#### 2.2.2. Nursing methods

##### 2.2.2.1. Control group

The control group received conventional emergency care as follows: upon the patient’s arrival at the hospital emergency department, the emergency nurse promptly conducted a rapid assessment of the patient’s condition and carried out corresponding emergency treatments according to medical orders. Routine examinations such as CT scans, complete blood count, liver and kidney function tests, and coagulation profiles were also completed. The patient’s vital signs were monitored, intravenous access was established, and all preparatory work for thrombolysis was thoroughly implemented. After the patient successfully completed thrombolysis, they were transferred to the Neurology Department, and handover procedures were completed with the neurology nurse.

##### 2.2.2.2. Observation group

The observation group received a modified emergency nursing pathway under the shared decision-making model, as follows:

(1) Information exchange: Emergency and medical staff provided patients and their families with comprehensive information on various aspects, including basic disease knowledge, advantages and disadvantages of emergency treatment methods, potential risks, and prognosis. They also assessed the needs, preferences, expectations, and values of the patients and families, establishing a relationship of respect and trust with them.
(2) Risk assessment: Based on the patient’s actual situation, including time of onset, comorbidities, severity of condition, and allergy history, emergency and medical staff identified the risks of thrombolytic therapy for the patient, such as infection, bleeding, and secondary infarction. They evaluated the effectiveness and feasibility of thrombolytic therapy and provided reasonable recommendations.
(3) Value consideration: Patients and families, considering their own values – including psychological stress, economic burden, and quality of life – fully expressed their wishes and choices regarding thrombolytic therapy and engaged in negotiation and communication with emergency and medical staff.
(4) Option selection: On the premise of fully respecting and understanding the wishes and choices of the patient and family, the emergency staff, medical staff, patient, and family jointly agreed on the optimal emergency care plan during the thrombolytic therapy period. Informed consent was signed, after which emergency and related nursing work were strictly carried out according to the plan.
(5) Plan implementation:
  ① After admission: The emergency triage received and assessed the patient’s condition, urgently collecting medical history, performing physical examinations, and assessing neurological function. Simultaneously, vital signs were immediately monitored, blood samples were collected and sent for testing, and CT examinations were performed. The stroke pathway was then activated, and a neurologist was consulted.
  ② Pre-thrombolysis preparation: The patient was promptly assessed for eligibility for thrombolysis. Upon completion of the CT scan, the patient proceeded directly to the emergency rescue room to sign and confirm thrombolytic therapy. Subsequently, hospitalization was processed via the emergency rescue room to initiate thrombolysis. During this period, intravenous access was established, and the patient’s vital signs were monitored.
  ③ During thrombolysis: Throughout the entire thrombolysis process, drug preparation and administration were completed accurately and promptly in strict accordance with medical orders. Vital signs were closely monitored, attention was paid to observing for bleeding in the patient’s skin, mucous membranes, gums, etc, and the patient’s reactions were noted. Any abnormalities were immediately reported to the physician.
  ④ Post-thrombolysis: After thrombolysis was completed, the patient needed to be promptly transferred to the neurology ward. Continuous cardiac monitoring was maintained, blood pressure fluctuations were noted, and monitoring for thrombolysis complications was carried out alongside predictive nursing interventions. The handover of the patient’s condition was completed.

### 2.3. Observation indicators

The total effectiveness rate, triage time, thrombolysis waiting time, rescue time, complication rate, National Institutes of Health Stroke Scale (NIHSS) scores, and Activities of Daily Living (ADL) scale scores before and after thrombolysis were observed and compared between the 2 groups. The modified Rankin Scale (mRS) scores at discharge were obtained through follow-up. The total effectiveness rate was based on NIHSS reduction from baseline to discharge. Complications were defined as events occurring from thrombolysis initiation to discharge. mRS was assessed at discharge.

1. Total effectiveness rate: Efficacy in both groups was assessed based on NIHSS scores. Outcomes were categorized as follows: “Basically Cured” for a reduction in NIHSS score > 90% compared to pretreatment; “Markedly Effective” for a reduction of 45% to 90%; “Effective” for a reduction of 18% to 44%; and “Ineffective” for a reduction of <18% or an increase.^[8]^ Total Effectiveness Rate = (Number of Basically Cured + Markedly Effective + Effective cases)/ Total number × 100%.
2. Treatment efficiency: This primarily included several indicators: triage time, thrombolysis waiting time, and rescue time for both groups.
3. Complications: These mainly included subcutaneous ecchymosis, gingival bleeding, epistaxis, digestive system bleeding, urinary system bleeding, intracranial hemorrhage, etc. The proportion of patients experiencing complications in each group was calculated.
4. NIHSS score: The NIHSS scores of both groups were assessed before and after thrombolysis. The total score ranges from 0 to 42, with a higher score indicating more severe neurological impairment.^[9]^
5. ADL score: The ADL scores of both groups were assessed before and after thrombolysis. The total score ranges from 0 to 100, with a higher score indicating better daily living activity ability.^[10]^
6. mRS score: Follow-up was conducted for patients in both groups to assess their mRS scores at discharge. The total score ranges from 0 to 6, where a score ≤2 represents a favorable prognosis, and a score ≥3 represents an unfavorable prognosis.^[11]^

### 2.4. Statistical methods

Statistical analysis was performed using SPSS 25.0 software. Count data were expressed as (n [%]) and analyzed using the chi-square (*χ*^2^) test. Measurement data that conformed to a normal distribution were expressed as (mean ± standard deviation) and analyzed using the *t* test. Data not conforming to a normal distribution were subjected to variable transformation to achieve normality before statistical analysis. A *P*-value < .05 was considered statistically significant. Data completeness was checked before statistical analysis. In this study, no significant missing data were identified for the primary outcome variables.

## 3. Results

### 3.1. Comparison of baseline characteristics between the 2 groups

No statistically significant differences were observed in baseline characteristics such as gender, age, smoking history, drinking history, comorbidities, time from onset to hospital admission, lesion location, and disease type between the 2 groups (*P* > .05). Details are provided in Table 1.

**Table 1:** Comparison of baseline data between the 2 groups.

Indicator	Observation group (n = 40)	Control group (n = 40)	*χ*^2^/*t*	*P*-value
Gender (n [%])			1.398	.237
Male	29 (72.50)	24 (60.00)		
Female	11 (27.50)	16 (40.00)		
Age (mean ± SD, yr)	67.25 ± 9.73	67.98 ± 12.08	0.298	.767
Smoking history (n [%])	10 (25.00)	12 (30.00)	0.251	.617
Drinking history (n [%])	8 (20.00)	7 (17.50)	0.082	.775
Comorbidities (n [%])				
Hypertension	6 (15.00)	5 (12.50)	0.105	.745
Diabetes	5 (12.50)	4 (10.00)	0.125	.723
Onset to admission time (mean ± SD, h)	3.48 ± 1.05	3.65 ± 1.20	0.674	.502
Lesion location (n [%])			1.515	.679
Cerebral artery	35 (87.50)	34 (85.00)		
Basal ganglia	1 (2.50)	0 (0.00)		
Carotid artery	3 (7.50)	5 (12.50)		
Vertebral artery	1 (2.50)	1 (2.50)		
Stroke subtype (n [%])			0.054	.816
Large artery atherosclerosis	25 (62.50)	26 (65.00)		
Small artery occlusion	15 (37.50)	14 (35.00)		

SD = standard deviation.

### 3.2. Comparison of total effectiveness rate between the 2 groups

In the observation group, the number of patients categorized as basically cured, markedly effective, effective, and ineffective was 10, 15, 13, and 2, respectively, resulting in a total effectiveness rate of 95.00%. In the control group, the corresponding numbers were 8, 12, 11, and 9, yielding a total effectiveness rate of 77.50%. The total effectiveness rate of the observation group was significantly higher than that of the control group, and the difference between the groups was statistically significant (*P* < .05). Details are presented in Table 2.

**Table 2:** Comparison of total effective rate between the 2 groups (n [%]).

Group	Basically cured	Markedly effective	Effective	Ineffective	Total effective rate
Observation (n = 40)	10 (25.00)	15 (37.50)	13 (32.50)	2 (5.00)	38 (95.00)
Control (n = 40)	8 (20.00)	12 (30.00)	11 (27.50)	9 (22.50)	31 (77.50)
*χ* ^2^	–	–	–	–	5.165
*P*	–	–	–	–	.023

### 3.3. Comparison of treatment efficiency indicators between the 2 groups

The triage time (0.59 ± 0.15 min vs 2.80 ± 0.62 min), thrombolysis waiting time (40.20 ± 4.26 min vs 50.38 ± 4.50 min), and rescue time (39.60 ± 5.24 min vs 57.20 ± 2.74 min) in the observation group were significantly shorter compared to the control group. The differences between the groups were statistically significant (*P* < .05). Details are provided in Table 3.

**Table 3:** Comparison of treatment efficiency indicators between the 2 groups (mean ± SD, min).

Group	Triage time	Thrombolysis waiting time	Rescue time
Observation (n = 40)	0.59 ± 0.15	40.20 ± 4.26	39.60 ± 5.24
Control (n = 40)	2.80 ± 0.62	50.38 ± 4.50	57.20 ± 2.74
*t*-value	21.912	10.39	18.825
*P*-value	<.001	<.001	<.001

SD = standard deviation.

### 3.4. Comparison of complication incidence between the 2 groups

In the observation group, 1 case each of subcutaneous ecchymosis and gingival bleeding occurred, resulting in a complication rate of 5.00%. In the control group, there were 3 cases of subcutaneous ecchymosis, 2 cases of gingival bleeding, 2 cases of epistaxis, 1 case of digestive system bleeding, and 1 case of urinary system bleeding, as well as 1 case of intracranial hemorrhage, resulting in a complication rate of 22.50%. The complication rate in the observation group was significantly lower than that in the control group, and the difference between the groups was statistically significant (*P* < .05). Details are provided in Table 4.

**Table 4:** Comparison of complication incidence between the 2 groups (n [%]).

Group	Subcutaneous ecchymosis	Gingival bleeding	Epistaxis	Digestive and urinary system bleeding	Intracranial hemorrhage	Total
Observation (n = 40)	1 (2.50)	1 (2.50)	0 (0.00)	0 (0.00)	0 (0.00)	2 (5.00)
Control (n = 40)	3 (7.50)	2 (5.00)	2 (5.00)	1 (2.50)	1 (2.50)	9 (22.50)
*χ* ^2^	–	–	–	–	–	5.165
*P*-value	–	–	–	–	–	.023

### 3.5. Comparison of NIHSS, ADL, and MRS scores between the 2 groups

Before thrombolysis, there were no statistically significant differences between the 2 groups in NIHSS scores (5.90 ± 0.60 vs 6.12 ± 0.68) or ADL scores (40.48 ± 5.14 vs 40.76 ± 5.28; *P* > .05). After thrombolysis, the NIHSS score in the observation group (3.13 ± 0.48) was significantly lower than that in the control group (5.00 ± 1.12), while the ADL score in the observation group (66.32 ± 6.75) was significantly higher than that in the control group (56.80 ± 6.12). Furthermore, the mRS score at discharge in the observation group (1.18 ± 0.20) was significantly lower than that in the control group (1.91 ± 0.48). All these intergroup differences were statistically significant (*P* < .05). Details are provided in Table 5.

**Table 5:** Comparison of NIHSS, ADL, and MRS scores between the 2 groups (mean ± SD, points).

Group	NIHSS score		ADL score		mRS score at discharge
	Pre-thrombolysis	Post-thrombolysis	Pre-thrombolysis	Post-thrombolysis	
Observation (n = 40)	5.90 ± 0.60	3.13 ± 0.48	40.48 ± 5.14	66.32 ± 6.75	1.18 ± 0.20
Control (n = 40)	6.12 ± 0.68	5.00 ± 1.12	40.76 ± 5.28	56.80 ± 6.12	1.91 ± 0.48

ADL = activities of daily living, mRS = modified Rankin Scale, NIHSS = National Institutes of Health Stroke Scale, SD = standard deviation.

### 3.6. Adjusted analyses

After adjustment for age, sex, time from symptom onset to hospital admission, baseline NIHSS score, hypertension, and diabetes, the modified emergency nursing pathway based on the shared decision-making model remained significantly associated with improved clinical and process outcomes.

Specifically, the observation group showed a higher total effectiveness rate (adjusted OR = 5.42, 95% CI: 1.68–17.51, *P* = .004) and a lower complication rate (adjusted OR = 0.18, 95% CI: 0.03–0.98, *P* = .047). In addition, triage time, thrombolysis waiting time, and rescue time were significantly shorter in the observation group. The post-thrombolysis NIHSS score was significantly lower, whereas the ADL score was significantly higher. The mRS score at discharge was also significantly lower (all *P* < .05). These findings indicate that the observed benefits of the modified emergency nursing pathway were independent of baseline characteristics (Table 6).

**Table 6:** Adjusted analyses of clinical and process outcomes.

Outcome	Adjusted effect (95% CI)	*P*-value
Total effectiveness rate	OR = 5.42 (1.68–17.51)	.004
Complications	OR = 0.18 (0.03–0.98)	.047
Triage time (min)	β = −2.05 (−2.30 to −1.80)	<.001
Thrombolysis waiting time (min)	β = −9.85 (−12.10 to −7.60)	<.001
Rescue time (min)	β = −17.20 (−19.80 to −14.60)	<.001
Post-thrombolysis NIHSS	β = −1.75 (−2.10 to −1.40)	<.001
Post-thrombolysis ADL	β = 8.55 (6.50–11.40)	<.001
mRS at discharge	β = −0.65 (−0.85 to −0.45)	<.001

ADL = activities of daily living, mRS = modified Rankin Scale, NIHSS = National Institutes of Health Stroke Scale.

## 4. Discussion

Acute ischemic stroke is characterized by sudden onset and rapid progression. If clinical treatment is not timely, it can easily lead to functional impairment, resulting in severe consequences such as disability or even death.^[12]^ Intravenous thrombolysis is one of the common clinical treatments for this condition. Among the agents used, alteplase, a type of thrombolytic drug, is an activator related to plasminogen. After administration, it can bind to lysine residues, fibrin, etc, and activate the plasminogen bound to fibrin, converting it into plasmin. This promotes the breakdown of fibrin into degradation products, thereby preventing platelet aggregation and reducing thrombus formation.^[13]^ Relevant studies have shown that early thrombolysis with alteplase can improve patient outcomes and prognosis.^[14-16]^ However, traditional emergency care often faces issues such as disjointed processes, prolonged patient transfer times, and inadequate monitoring, leading to delays in patient treatment. Therefore, actively exploring more scientific and effective emergency nursing methods is crucial for improving the efficiency of thrombolysis therapy in patients with acute ischemic stroke.

Research indicates that the emergency nursing process directly impacts the quality of acute ischemic stroke care.^[17]^ Ferri^[18]^ found that modifying the emergency nursing pathway for use during thrombolysis in acute ischemic stroke can improve patient outcomes. This study similarly demonstrated that the modified emergency nursing pathway based on the shared decision-making model resulted in a higher total effectiveness rate in acute ischemic stroke patients, which is largely consistent with the aforementioned findings. The primary reason is that the shared decision-making model involves inviting patients and their families to participate in emergency decision-making, highly respecting their autonomy. This enhances the sense of involvement and responsibility among patients and families, thereby improving their compliance and satisfaction. It also strengthens the professionalism and humanistic care of medical staff, increasing their trust and satisfaction, ultimately enhancing the effectiveness and feasibility of the emergency plan, reducing the risk of treatment failure, and improving emergency outcomes.^[19,20]^

The observation group had shorter triage time, thrombolysis waiting time, and rescue time compared to the control group (*P* < .05), indicating that the modified emergency nursing pathway based on the shared decision-making model can improve treatment efficiency. This is because the observation group used the shared decision-making model as a foundation, dividing emergency care into stages: post-admission, pre-thrombolysis preparation, during thrombolysis, and post-thrombolysis. This patient-centered approach, combined with hospital conditions, maximally improved and optimized the emergency nursing pathway. This nursing protocol effectively integrates emergency resources, standardizes the emergency process, clarifies responsibilities, enhances collaboration, reduces or avoids delays, and secures more treatment time for patients, thereby improving emergency efficiency.^[21,22]^

The complication rate in the observation group was lower than that in the control group (*P* < .05), indicating that the modified emergency nursing pathway based on the shared decision-making model can reduce related complications. The likely reason is that this nursing plan is tailored to the patient’s actual condition. It includes post-thrombolysis blood pressure monitoring, vigilance for thrombolysis-related complications, and predictive nursing interventions, which can effectively reduce unnecessary complications.^[23]^

The NIHSS score after thrombolysis in the observation group was lower than that in the control group, the ADL score was higher, and the mRS score at discharge was lower (*P* < .05). This reflects that the modified emergency nursing pathway based on the shared decision-making model can improve patients’ neurological function and prognosis and enhance their daily living abilities. The reason is that the nursing measures in the observation group effectively address the shortcomings of traditional emergency care, ensuring a seamless connection between various emergency steps. Through close cooperation between medical staff, patients, and their families, it provides scientific, standardized, and efficient emergency nursing, thereby helping to improve patient conditions and achieve desirable outcomes.^[24,25]^ Furthermore, the modified emergency nursing pathway based on the shared decision-making model has potential applicability in different healthcare environments. Even in hospitals with relatively limited resources, optimizing emergency processes, strengthening interdisciplinary cooperation, and improving communication with patients and their families may significantly enhance treatment efficiency and clinical outcomes. Therefore, this model may provide a practical reference for improving stroke emergency care systems in various healthcare settings.

## 5. Limitations and future directions

Although this study has achieved the aforementioned results, it still has limitations. For instance, it is a single-center study with a relatively small sample size and is only applicable to the ischemic stroke population receiving thrombolysis. The representativeness of the findings needs further validation in future studies. Due to time constraints, the study lacks long-term follow-up data on acute ischemic stroke patients. Furthermore, it did not analyze the impact of the nursing protocol on aspects such as inflammatory response and oxidative stress in patients. The specific mechanisms of action and molecular targets of the nursing protocol remain unclear. Because this was a retrospective observational study, residual confounding cannot be completely excluded despite multivariable adjustment.

Therefore, future research should aim to increase the sample size and conduct multi-center, long-term randomized controlled trials to verify the long-term efficacy and safety of this nursing protocol. It is also necessary to collect data on changes in inflammatory factors and oxidative stress indicators in patients before and after the intervention. Continuous improvement of emergency nursing methods is essential, actively working towards establishing a standardized emergency nursing strategy for thrombolysis patients in the local region, thereby enhancing the scientific rigor and standardization of specialized nursing measures within the regional scope.

## 6. Conclusion

In conclusion, the application of the modified emergency nursing pathway based on the shared decision-making model during alteplase thrombolysis for acute ischemic stroke can improve treatment efficiency and clinical outcomes, reduce the incidence of complications, enhance neurological function and prognosis, and increase the ability in daily activities.

## Author contributions

**Conceptualization:** Haixia Liu.

**Data curation:** Haixia Liu.

**Formal analysis:** Haixia Liu.

**Funding acquisition:** Haixia Liu.

**Investigation:** Haixia Liu.

**Writing – original draft:** Haixia Liu.

**Writing – review & editing:** Haixia Liu.
